# Time to jump on the bandwagon: the Journal of Cachexia, Sarcopenia and Muscle in 2018

**DOI:** 10.1002/jcsm.12356

**Published:** 2018-10-11

**Authors:** Stephan von Haehling, Markus S. Anker, Nicole Ebner, Stefan D. Anker

**Affiliations:** ^1^ Department of Cardiology and Pneumology University of Göttingen Medical School Göttingen Germany; ^2^ Division of Cardiology and Metabolism, Department of Cardiology Charité‐Universitätsmedizin Berlin Berlin Germany; ^3^ Berlin‐Brandenburg Center for Regenerative Therapies (BCRT) Berlin Germany; ^4^ DZHK (German Centre for Cardiovascular Research), partner site: Department of Cardiology Campus Benjamin Franklin, Charité‐Universitätsmedizin Berlin Berlin Germany; ^5^ Department of Cardiology (CVK) Charité‐Universitätsmedizin Berlin Berlin Germany

The *Journal of Cachexia, Sarcopenia and Muscle* (JCSM) was launched in September 2010, which means that we are publishing in the 9th year. Looking at PubMed, we found 445 entries when we searched the database on 7 September 2018. The overall number of publications has increased along with the number of issues from 4 to 5 in 2016 and again to 6 per year in 2017. Quite interestingly, though, the impact factor, published by Thomson Scientific in June this year, has also risen from 9.697 to 12.511. Of course, this is the 2017 impact factor, because the 2018 impact factor will become available only next year. It often comes as a surprise that the impact factor publication always bears the previous year. This has to do with the fact that it is calculated based on the items published and cited in the previous two full years. For JCSM, this means that the impact factor 2017 was calculated based on items cited and published in 2016 and 2017, but not on 2018 data as these are not available in full in the middle of the year. Looking at content and using the search term ‘J Cachexia Sarcopenia Muscle [jo] AND muscle [ti]’, we found 191 items published in JCSM that have the word ‘muscle’ in the manuscript title. For cachexia and sarcopenia, these numbers are 140 and 66, respectively.

JCSM is currently listed in Thomson Scientific only in two categories, in ‘Geriatrics and Gerontology’ and in ‘Medicine, General and Internal’ where it is ranked number 1 and number 8, respectively. We feel that listing in ‘Nutrition and Dietetics’ would also be appropriate, but currently, this is not the case (*Tables* 1, 2, 3). The best papers published in JCSM are listed in *Tables* 4, 5, 6, 7.^1^

**Table 1:** Top 10 journals in the field ‘Medicine: General & Internal’ 2017

Rank	Full journal title	2017 Journal impact factor	Citable items published in 2015 and 2016	Issues per year
1	NEW ENGLAND JOURNAL OF MEDICINE	79.258	670	52
2	LANCET	53.254	645	51
3	JAMA‐JOURNAL OF THE AMERICAN MEDICAL ASSOCIATION	47.661	410	48
4	BMJ‐British Medical Journal	23.259	448	52
5	JAMA Internal Medicine	19.989	275	12
6	ANNALS OF INTERNAL MEDICINE	19.384	302	12
7	Nature Reviews Disease Primers	16.071	84	Continuous
8	Journal of Cachexia Sarcopenia and Muscle	12.511	88	6
9	PLOS MEDICINE	11.675	286	52
10	BMC Medicine	9.088	398	Continuous

**Table 2:** Top 10 journals in the field ‘Nutrition & Dietetics’ 2017, where the Journal of Cachexia, Sarcopenia and Muscle is officially not listed

Rank	Full journal title	2017 Journal impact factor	Citable items published in 2015 and 2016	Issues per year
1	Annual Review of Nutrition	8.886	44	1
2	PROGRESS IN LIPID RESEARCH	8.435	69	4
3	Advances in Nutrition	6.853	177	6
4	AMERICAN JOURNAL OF CLINICAL NUTRITION	6.549	649	12
5	CRITICAL REVIEWS IN FOOD SCIENCE AND NUTRITION	6.015	334	12
6	NUTRITION REVIEWS	5.788	132	12
7	International Journal of Behavioral Nutrition and Physical Activity	5.548	292	Continuous
8	CLINICAL NUTRITION	5.496	375	6
9	PROCEEDINGS OF THE NUTRITION SOCIETY	5.347	118	5
10	INTERNATIONAL JOURNAL OF OBESITY	5.151	503	12

**Table 3:** Top 10 journals in the field ‘Geriatrics & Gerontology’ 2017

Rank	Full journal title	2017 Journal impact factor	Citable items published in 2015 and 2016	Issues per year
1	Journal of Cachexia Sarcopenia and Muscle	12.511	88	6
2	AGEING RESEARCH REVIEWS	8.973	146	8
3	AGING CELL	7.627	225	6
4	Journal of the American Medical Directors Association	5.325	388	12
5	Aging‐US	5.179	296	12
6	Aging and Disease	5.058	104	6
7	JOURNALS OF GERONTOLOGY SERIES A‐BIOLOGICAL SCIENCES AND MEDICAL SCIENCES	4.902	419	12
8	NEUROBIOLOGY OF AGING	4.454	738	12
9	JOURNAL OF THE AMERICAN GERIATRICS SOCIETY	4.155	627	12
10	Immunity & Ageing	4.019	53	Continuous

**Table 4:** Top 10 of best cited original articles since first publication of the Journal of Cachexia, Sarcopenia and Muscle

	First author	Title	Type	Year	Cites all time	Reference
1	Dalton	The selective androgen receptor modulator GTx‐024 (enobosarm) improves lean body mass and physical function in healthy elderly men and postmenopausal women: results of a double‐blind, placebo‐controlled phase II trial	Article	2011	135	3
2	Cesari	Biomarkers of sarcopenia in clinical trials‐recommendations from the International Working Group on Sarcopenia	Article	2012	99	4
3	Lainscak	Body mass index and prognosis in patients hospitalized with acute exacerbation of chronic obstructive pulmonary disease	Article	2011	69	5
4	Malmstrom	SARC‐F: a symptom score to predict persons with sarcopenia at risk for poor functional outcomes	Article	2016	66	6
5	Rozentryt	The effects of a high‐caloric protein‐rich oral nutritional supplement in patients with chronic heart failure and cachexia on quality of life, body composition, and inflammation markers: a randomized, double‐blind pilot study	Article	2010	64	7
6	Argiles	The cachexia score (CASCO): a new tool for staging cachectic cancer patients	Article	2011	56	8
7	Busquets	Myostatin blockage using actRIIB antagonism in mice bearing the Lewis lung carcinoma results in the improvement of muscle wasting and physical performance	Article	2012	55	9
8	Kilgour	Cancer‐related fatigue: the impact of skeletal muscle mass and strength in patients with advanced cancer	Article	2010	46	10
9	Chen	Ghrelin prevents tumour‐ and cisplatin‐induced muscle wasting: characterization of multiple mechanisms involved	Article	2015	45	11
10	Stephens	Intramyocellular lipid droplets increase with progression of cachexia in cancer patients	Article	2011	43	12

**Table 5:** Top 30 of best cited articles published 2015 in the Journal of Cachexia, Sarcopenia and Muscle and cited in 2017

	First author	Title	Type	Cites in 2017	Reference
1	von Haehling	Ethical guidelines for publishing in the Journal of Cachexia, Sarcopenia and Muscle: update 2015	Editorial	73	13
2	Bowen	Skeletal muscle wasting in cachexia and sarcopenia: molecular pathophysiology and impact of exercise training	Review	38	14
3	Calvani	Biomarkers for physical frailty and sarcopenia: state of the science and future developments	Review	27	15
4	Ezeoke	Pathophysiology of anorexia in the cancer cachexia syndrome	Review	26	16
5	Chen	Ghrelin prevents tumour‐ and cisplatin‐induced muscle wasting: characterization of multiple mechanisms involved	Article	24	11
6	Drescher	Loss of muscle mass: current developments in cachexia and sarcopenia focused on biomarkers and treatment	Review	20	17
7	Sasso	A framework for prescription in exercise‐oncology research	Editorial	18	18
8	Fearon	Request for regulatory guidance for cancer cachexia intervention trials	Editorial	17	19
9	Anker	Cachexia: a nutritional syndrome?	Editorial	15	20
10	Mangner	Skeletal muscle alterations in chronic heart failure: differential effects on quadriceps and diaphragm	Article	14	21
11	Grande	Exercise for cancer cachexia in adults: executive summary of a Cochrane Collaboration systematic review	Review	13	22
12	Cooper	Understanding and managing cancer‐related weight loss and anorexia: insights from a systematic review of qualitative research	Review	12	23
13	Dupuy	Searching for a relevant definition of sarcopenia: results from the cross‐sectional EPIDOS study	Article	11	24
14	Stephens	Evaluating potential biomarkers of cachexia and survival in skeletal muscle of upper gastrointestinal cancer patients	Article	11	25
15	Matsuo	Fibronectin type III domain containing 5 expression in skeletal muscle in chronic heart failure‐relevance of inflammatory cytokines	Article	11	26
16	Lerner	Plasma growth differentiation factor 15 is associated with weight loss and mortality in cancer patients	Article	11	27
17	Morley	Rapid screening for sarcopenia	Editorial	11	28
18	Anker	Evidence for partial pharmaceutical reversal of the cancer anorexia‐cachexia syndrome: the case of anamorelin	Editorial	11	29
19	Gu	Nutritional screening is strongly associated with overall survival in patients treated with targeted agents for metastatic renal cell carcinoma	Article	10	30
20	Dev	Hypermetabolism and symptom burden in advanced cancer patients evaluated in a cachexia clinic	Article	10	31
21	Dwarkasing	Differences in food intake of tumour‐bearing cachectic mice are associated with hypothalamic serotonin signalling	Article	9	32
22	Gielen	Endocrine determinants of incident sarcopenia in middle‐aged and elderly European men	Article	8	33
23	van Dijk	Effects of oral meal feeding on whole body protein breakdown and protein synthesis in cachectic pancreatic cancer patients	Article	8	34
24	Wakabayashi	Skeletal muscle mass is associated with severe dysphagia in cancer patients	Article	8	35
25	Kob	Gender‐specific differences in the development of sarcopenia in the rodent model of the ageing high‐fat rat	Article	8	36
26	Marino	Activin‐beta(C) modulates cachexia by repressing the ubiquitin‐proteasome and autophagic degradation pathways	Article	8	37
27	van Norren	Behavioural changes are a major contributing factor in the reduction of sarcopenia in caloric‐restricted ageing mice	Article	7	38
28	Haruta	One‐year intranasal application of growth hormone releasing peptide‐2 improves body weight and hypoglycemia in a severely emaciated anorexia nervosa patient	Article	6	39
29	Faber	Improved body weight and performance status and reduced serum PGE(2) levels after nutritional intervention with a specific medical food in newly diagnosed patients with esophageal cancer or adenocarcinoma of the gastro‐esophageal junction	Article	5	40
30	Moryoussef	Reversible sarcopenia in patients with gastrointestinal stromal tumor treated with imatinib	Article	5	41

**Table 6:** Top 30 of best cited articles published 2016 in the Journal of Cachexia, Sarcopenia and Muscle and cited in 2017

	First author	Title	Type	Cites in 2017	Reference
1	Malmstrom	SARC‐F: a symptom score to predict persons with sarcopenia at risk for poor functional outcomes	Article	31	5
2	Coats	Espindolol for the treatment and prevention of cachexia in patients with stage III/IV non‐small cell lung cancer or colorectal cancer: a randomized, double‐blind, placebo‐controlled, international multicentre phase II study (the ACT‐ONE trial)	Article	24	42
3	Montano‐Loza	Sarcopenic obesity and myosteatosis are associated with higher mortality in patients with cirrhosis	Article	18	43
4	Brown	Sarcopenia and mortality among a population‐based sample of community‐dwelling older adults	Article	18	44
5	Rutten	Loss of skeletal muscle during neoadjuvant chemotherapy is related to decreased survival in ovarian cancer patients	Article	17	45
6	Tyrovolas	Factors associated with skeletal muscle mass, sarcopenia, and sarcopenic obesity in older adults: a multi‐continent study	Article	16	46
7	Sanders	Cachexia in chronic obstructive pulmonary disease: new insights and therapeutic perspective	Review	16	47
8	Loncar	Cardiac cachexia: hic et nunc	Review	15	48
9	von Haehling	Prevalence and clinical impact of cachexia in chronic illness in Europe, USA, and Japan: facts and numbers update 2016	Editorial	15	49
10	Sente	Adiponectin resistance in skeletal muscle: pathophysiological implications in chronic heart failure	Review	14	50
11	Leong	Reference ranges of handgrip strength from 125,462 healthy adults in 21 countries: a prospective urban rural epidemiologic (PURE) study	Article	12	51
12	Sakuma	p62/SQSTM1 but not LC3 is accumulated in sarcopenic muscle of mice	Article	12	52
13	Pinto	Impact of creatine supplementation in combination with resistance training on lean mass in the elderly	Article	12	53
14	Banach	Discussion around statin discontinuation in older adults and patients with wasting diseases	Editorial	12	54
15	Batista	Cachexia‐associated adipose tissue morphological rearrangement in gastrointestinal cancer patients	Article	11	55
16	Go	Prognostic impact of sarcopenia in patients with diffuse large B‐cell lymphoma treated with rituximab plus cyclophosphamide, doxorubicin, vincristine, and prednisone	Article	11	56
17	Anker	Welcome to the ICD‐10 code for sarcopenia	Editorial	11	57
18	Lainscak	ACT‐ONE‐ACTION at last on cancer cachexia by adapting a novel action beta‐blocker	Editorial	11	58
19	Barbosa‐Silva	Prevalence of sarcopenia among community‐dwelling elderly of a medium‐sized South American city: results of the COMO VAI? study	Article	10	59
20	Ferraro	Improvement of skeletal muscle performance in ageing by the metabolic modulator Trimetazidine	Article	10	60
21	Foong	Accelerometer‐determined physical activity, muscle mass, and leg strength in community‐dwelling older adults	Article	10	61
22	Lewis	Increased expression of H19/miR‐675 is associated with a low fat‐free mass index in patients with COPD	Article	10	62
23	Penna	Effect of the specific proteasome inhibitor bortezomib on cancer‐related muscle wasting	Article	10	63
24	Musolino	Megestrol acetate improves cardiac function in a model of cancer cachexia‐induced cardiomyopathy by autophagic modulation	Article	9	64
25	Berger	Dysfunction of respiratory muscles in critically ill patients on the intensive care unit	Review	9	65
26	de Vries	Patient‐centred physical therapy is (cost‐) effective in increasing physical activity and reducing frailty in older adults with mobility problems: a randomized controlled trial with 6 months follow‐up	Article	8	66
27	Patel	Growth differentiation factor‐15 is associated with muscle mass in chronic obstructive pulmonary disease and promotes muscle wasting in vivo	Article	8	67
28	Szulc	High risk of fall, poor physical function, and low grip strength in men with fracture—the STRAMBO study	Article	8	68
29	Lodka	Muscle RING‐finger 2 and 3 maintain striated‐muscle structure and function	Article	8	69
30	Nederveen	Skeletal muscle satellite cells are located at a closer proximity to capillaries in healthy young compared with older men	Article	7	70

**Table 7:** Top 30 of best cited articles published 2017 in the Journal of Cachexia, Sarcopenia and Muscle

	First author	Title	Type	Cites all time	Reference
1	Kalafateli	Malnutrition and sarcopenia predict post‐liver transplantation outcomes independently of the Model for End‐stage Liver Disease score	Article	28	71
2	Sahebkar	Curcumin: an effective adjunct in patients with statin‐associated muscle symptoms?	Review	18	72
3	van Dijk	Low skeletal muscle radiation attenuation and visceral adiposity are associated with overall survival and surgical site infections in patients with pancreatic cancer	Article	15	73
4	Mochamat	A systematic review on the role of vitamins, minerals, proteins, and other supplements for the treatment of cachexia in cancer: a European Palliative Care Research Centre cachexia project	Review	14	74
5	Snijders	Muscle fibre capillarization is a critical factor in muscle fibre hypertrophy during resistance exercise training in older men	Article	11	75
6	Calvani	Systemic inflammation, body composition, and physical performance in old community‐dwellers	Article	11	76
7	Boengler	Mitochondria and ageing: role in heart, skeletal muscle and adipose tissue	Review	11	77
8	Klassen	Muscle strength in breast cancer patients receiving different treatment regimes	Article	10	78
9	Kittiskulnam	Sarcopenia among patients receiving hemodialysis: weighing the evidence	Article	10	79
10	Cheung	Androgen deprivation causes selective deficits in the biomechanical leg muscle function of men during walking: a prospective case–control study	Article	10	80
11	Verzola	Toll‐like receptor 4 signalling mediates inflammation in skeletal muscle of patients with chronic kidney disease	Article	10	81
12	Solheim	A randomized phase II feasibility trial of a multimodal intervention for the management of cachexia in lung and pancreatic cancer	Article	9	82
13	Beaudart	Validation of the SarQoL (R), a specific health‐related quality of life questionnaire for Sarcopenia	Article	9	83
14	Chan	Integrated care for geriatric frailty and sarcopenia: a randomized control trial	Article	9	84
15	Holecek	Beta‐hydroxy‐beta‐methylbutyrate supplementation and skeletal muscle in healthy and muscle‐wasting conditions	Review	9	85
16	van de Bool	A randomized clinical trial investigating the efficacy of targeted nutrition as adjunct to exercise training in COPD	Article	8	86
17	Reijnierse	Assessment of maximal handgrip strength: how many attempts are needed?	Article	8	87
18	St‐Jean‐Pelletier	The impact of ageing, physical activity, and pre‐frailty on skeletal muscle phenotype, mitochondrial content, and intramyocellular lipids in men	Article	8	88
19	Dodds	Prevalence and incidence of sarcopenia in the very old: findings from the Newcastle 85+ Study	Article	8	89
20	dos Santos	Sarcopenia and physical independence in older adults: the independent and synergic role of muscle mass and muscle function	Article	8	90
21	van Vugt	A comparative study of software programmes for cross‐sectional skeletal muscle and adipose tissue measurements on abdominal computed tomography scans of rectal cancer patients	Article	8	91
22	Molfino	Cancer anorexia: hypothalamic activity and its association with inflammation and appetite‐regulating peptides in lung cancer	Article	8	92
23	Goossens	Premorbid obesity, but not nutrition, prevents critical illness‐induced muscle wasting and weakness	Article	8	93
24	Lipina	Lipid modulation of skeletal muscle mass and function	Review	8	94
25	Clark	Effect of beta‐adrenergic blockade with carvedilol on cachexia in severe chronic heart failure: results from the COPERNICUS trial	Article	7	95
26	Rutten	Psoas muscle area is not representative of total skeletal muscle area in the assessment of sarcopenia in ovarian cancer	Article	7	96
27	Johns	New genetic signatures associated with cancer cachexia as defined by low skeletal muscle index and weight loss	Article	7	97
28	Gonzalez‐Freire	The Human Skeletal Muscle Proteome Project: a reappraisal of the current literature	Review	7	98
29	Morley	Anorexia of ageing: a key component in the pathogenesis of both sarcopenia and cachexia	Editorial	7	99
30	Baracos	Psoas as a sentinel muscle for sarcopenia: a flawed premise	Editorial	7	100

Looking at JCSM as the mother, there are now two daughters that have started to flourish: JCSM—Clinical Reports (http://www.jcsm-clinical-reports.info) started in July 2016 and is dedicated to clinical reports in the strictest sense of the word, i.e. original and review papers from the clinical field of wasting disorders in the broadest sense including case reports. The other one, JCSM—Rapid Communications (http://www.jcsm-rapid-communications.info), was launched a little later in January this year and is supposed to publish scientific papers from a very broad field including original and review papers from clinical as well as basic science groups. It is interesting to see how rapidly both journals are growing, underlining the need for more than one journal in the area of body wasting that can still be considered a niche. Of course, we welcome submissions to all three journals.

Finally, we would like to draw your attention to the up‐coming Cachexia Conference, to be held in Maastricht, the Netherlands, from December 7 to 9, 2018. The conference remains a source of stimulating ideas and exchange between clinicans and researchers in the field of cachexia and wasting. More information is available at http://society-scwd.org.

## Conflict of interest

None declared.
